# Single-stranded DNA binding protein Ssbp3 induces differentiation of mouse embryonic stem cells into trophoblast-like cells

**DOI:** 10.1186/s13287-016-0340-1

**Published:** 2016-05-28

**Authors:** Jifeng Liu, Xinlong Luo, Yanli Xu, Junjie Gu, Fan Tang, Ying Jin, Hui Li

**Affiliations:** Laboratory of Molecular Developmental Biology, Shanghai Jiao Tong University School of Medicine, Shanghai, 200025 China; Key Laboratory of Stem Cell Biology, Institute of Health Sciences, Shanghai Institutes for Biological Sciences, Chinese Academy of Sciences, Shanghai Jiao Tong University School of Medicine, New Life Science Building A, Room 1328, 320 Yue Yang Road, Shanghai, 200032 China; Present address: KU Leuven Department of Development and Regeneration, Stem Cell Institute Leuven, Herestraat 49, 3000, Leuven, Belgium

**Keywords:** Ssbp3, Mouse embryonic stem cells, Trophoblast, Differentiation

## Abstract

**Background:**

Intrinsic factors and extrinsic signals which control unlimited self-renewal and developmental pluripotency in embryonic stem cells (ESCs) have been extensively investigated. However, a much smaller number of factors involved in extra-embryonic trophoblast differentiation from ESCs have been studied. In this study, we investigated the role of the single-stranded DNA binding protein, Ssbp3, for the induction of trophoblast-like differentiation from mouse ESCs.

**Methods:**

Gain- and loss-of-function experiments were carried out through overexpression or knockdown of Ssbp3 in mouse ESCs under self-renewal culture conditions. Expression levels of pluripotency and lineage markers were detected by real-time quantitative reverse-transcription polymerase chain reaction (qRT-PCR) analyses. The global gene expression profile in Ssbp3-overexpressing cells was determined by affymetrix microarray. Gene ontology and pathway terms were analyzed and further validated by qRT-PCR and Western blotting. The methylation status of the Elf5 promoter in Ssbp3-overexpressing cells was detected by bisulfite sequencing. The trophoblast-like phenotype induced by Ssbp3 was also evaluated by teratoma formation and early embryo injection assays.

**Results:**

Forced expression of Ssbp3 in mouse ESCs upregulated expression levels of lineage-associated genes, with trophoblast cell markers being the highest. In contrast, depletion of Ssbp3 attenuated the expression of trophoblast lineage marker genes induced by downregulation of Oct4 or treatment with BMP4 and bFGF in ESCs. Interestingly, global gene expression profiling analysis indicated that Ssbp3 overexpression did not significantly alter the transcript levels of pluripotency-associated transcription factors. Instead, Ssbp3 promoted the expression of early trophectoderm transcription factors such as Cdx2 and activated MAPK/Erk1/2 and TGF-β pathways. Furthermore, overexpression of Ssbp3 reduced the methylation level of the Elf5 promoter and promoted the generation of teratomas with internal hemorrhage, indicative of the presence of trophoblast cells.

**Conclusions:**

This study identifies Ssbp3, a single-stranded DNA binding protein, as a regulator for mouse ESCs to differentiate into trophoblast-like cells. This finding is helpful to understand the regulatory networks for ESC differentiation into extra-embryonic lineages.

**Electronic supplementary material:**

The online version of this article (doi:10.1186/s13287-016-0340-1) contains supplementary material, which is available to authorized users.

## Background

The formation of the early blastocyst represents the first lineage specification of mammalian embryos. At this stage, the mammalian embryo forms an internal cavity, and contains two types of cells: the inner cell mass (ICM) growing on the interior and the monolayer of the trophectoderm (TE) growing on the exterior [1, 2]. The ICM possesses developmental pluripotency, and develops into the three germ layers as well as the extra-embryonic mesoderm portion of the placenta, while the TE gives rise to the extra-embryonic ectoderm portion of the placenta [3].

Embryonic stem cells (ESCs) and trophoblast stem cells (TSCs) are cell culture derivatives of the ICM and TE, respectively [4, 5]. These two stem cell types possess distinctly different abilities to self-renew and generate progenies, which are under the control of both extrinsic signals and intrinsic regulatory networks. Although derived from the ICM, ESCs can undergo *trans*-differentiation towards extra-embryonic trophoblast lineage via overexpression of TE master regulators, such as Cdx2 and Gata3 [6–8]. In contrast to the extensive studies on the molecular regulation of ESC self-renewal and pluripotency, only a few of the factors involved in extra-embryonic lineage differentiation of ESCs have been defined.

Ssbp3 (single-stranded DNA binding protein 3) belongs to a family of single-stranded DNA binding proteins which recognizes pyrimidine-rich single-stranded DNA and regulates transcription. It has two homologues, Ssbp2 and Ssbp4 [9]. In 1998, Bayarsaihan et al. discovered that Ssbp3 could bind a conserved sequence, containing pyrimidines almost exclusively, on one strand in chicken collagen α2 (I) promoter regions to regulate its expression [10]. Further studies revealed the function of Ssbp3 in regulating mouse head morphogenesis by protecting Ldb1 from Rlim-mediated ubiquitination in development. Ssbp3 null mice lacked the head structure from the anterior to the ear, accompanied by a small size and spine deformity [11–16]. However, the function of Ssbp3 in early embryonic and extra-embryonic development remains unknown.

In the present study, we report that Ssbp3 plays an important role in regulating mouse ESC differentiation to trophoblast-like cells. We show that the expression of Ssbp3 was increased in ESC differentiation models towards trophoblast lineages induced by either Oct4 downregulation or supplementation of bone morphogenetic protein (BMP)4 and basic fibroblast growth factor (bFGF). Forced expression of Ssbp3 substantially promoted differentiation. The cells overexpressing Ssbp3 had gene expression patterns resembling those of trophoblast cells and formed teratomas containing hemorrhages in vivo. In contrast, depletion of Ssbp3 compromised the induction of trophoblast differentiation from ESCs. Genome-wide gene expression analysis indicated that Ssbp3 overexpression in ESCs activated mitogen-activated protein kinase (MAPK)/extracellular signal-regulated kinase (Erk)1/2 and transforming growth factor (TGF)-β pathways, which are known to be critical for mouse trophoblast development [17–20], and induced the expression of trophoblast lineage-associated markers. Taken together, our results revealed that Ssbp3 played an important role in the control of ESCs to differentiate into trophoblast-like cells.

## Methods

### Cell culture

Mouse E14T and ZHBTc4 ESCs (kind gifts from Dr. Austin Smith) were cultured under feeder-free conditions in GMEM (Glasgow's minimum essential medium; Invitrogen) supplemented with 10 % fetal bovine serum (FBS; Hyclone), 1 mM sodium pyruvate (Sigma), 0.1 mM non-essential amino acids (NEAA; Invitrogen), 2 mM l-glutamine (Invitrogen), 100 μM β-mercaptoethanol (Invitrogen), 100 U/mL penicillin and 100 μg/mL streptomycin (Hyclone), and 1000 U/mL recombinant leukemia inhibitory factor (LIF; Chemicon).

Mouse TSCs (a kind gift from Dr. Shaorong Gao) were maintained in the medium containing 30 % TS medium and 70 % mouse embryonic fibroblast (MEF)-conditioned TS medium with 25 ng/mL human recombinant fibroblast growth factor 4 (FGF4; Invitrogen) and 1 μg/mL heparin (Sigma) in 0.1 % gelatin-coated dishes. In detail, the TSC medium based on RPMI 1640 (Invitrogen) was supplemented with 20 % FBS (Hyclone), 1 mM sodium pyruvate (Sigma), 2 mM l-glutamine (Invitrogen), 100 μM β-mercaptoethanol (Invitrogen), and 100 U/mL penicillin and 100 μg/mL streptomycin (Hyclone) [21].

MEF and 293T cells were cultured in Dulbecco's modified Eagle’s medium (DMEM; Invitrogen) with 10 % FBS (Hyclone), and 100 U/mL penicillin and 100 μg/mL streptomycin (Hyclone) [22].

The trophoblast induction medium was modified based on the TX medium [23], and contained the KnockOut DMEM/F-12 (Invitrogen), 64 μg/mL l-ascorbic acid-2-phosphate magnesium (ACC), insulin-transferrin-selenium (Life Technologies), 543 μg/mL NaHCO_3_ (Sigma), 1 μg/mL heparin (Sigma), 2 mM l-glutamine (Life Technologies), 100 U/mL penicillin and 100 μg/mL streptomycin (Hyclone), 10 ng/mL BMP4 (HUMANZYME), and 25 ng/mL bFGF (HUMANZYME).

### Plasmids and antibodies

The coding sequences of full length Ssbp3 [NCBI Reference Sequence: NM_023672.2], its truncations, and full length Cdx2 [NCBI Reference Sequence: NM_007673.3] were amplified by polymerase chain reaction (PCR) from complementary DNA (cDNA) of mouse ESCs or TSCs, respectively. PCR products were inserted into the pPyCAGIP expression vector (a kind gift from Dr. Ian Chambers). Primers used for gene cloning are listed in Additional file 1 (Table S1). Lentiviral vector pLKO.1-hygro (Addgene) was used to express short-hairpin RNAs (shRNAs) that targeted mouse Ssbp3, Cdx2, or Gata3 mRNA, respectively [24]. For lentiviral packaging, psPAX2 and pMD2.G vectors (kind gifts from Dr. Didier Trono) were used. shRNA targeting sequences are also listed in Additional file 1 (Table S1). The inserted sequences and ligation sites of all constructs were validated by DNA sequencing. Western blotting was carried out with primary antibodies against Ssbp3 (Genetex), Cdx2 (Biogenex), Oct4 (produced and affinity-purified in our laboratory), phospho-Erk1/2 (Cell Signaling Technology), total Erk1/2 (Cell Signaling Technology), and β-Tubulin (Sigma). For immunofluorescence staining, cells were incubated with primary antibody against Cdx2 (Biogenex) and then Cy3 conjugated goat anti-mouse secondary antibody (ProteinTech Group).

### Transfection and infection

Mouse ESCs were plated in six-well plates at a density of 1.0 × 10^5^ cells per well. Twenty-four hours later, cells were transfected with plasmids via Lipofectamine™2000 (Invitrogen), or infected with viral particles.

### Western blotting

Proteins extracted from the indicated cells were subjected to Western blotting with the amount of 100 μg proteins per lane, as described previously [25].

### Immunofluorescence staining

Mouse ESCs were plated in four-well plates containing coverslips at a density of 1.0 × 10^5^ cells per well and were transfected with an empty vector or an Ssbp3 expression plasmid on the second day, respectively. The cells were cultured at 37 °C for an additional 96 h. They were then fixed with 4 % paraformaldehyde for 15 min, permeabilized with 0.2 % Triton X-100 for 10 min, and blocked with 3 % bovine serum albumin (BSA) for 30 min. Next, cells were incubated with the primary anti-Cdx2 antibody (1:500 diluted) overnight at 4 °C. The next day, cells were incubated with the fluorescent Cy3-goat anti-mouse antibody (1:200 diluted) for 1 h at room temperature in the dark. The nuclei were further stained with 4',6-diamidino-2-phenylindole (DAPI; Invitrogen) for 3 min. Finally, coverslips were dried and affixed to slides using a fluorescent mounting medium [26].

### qRT-PCR analyses

Total RNA was extracted and transcribed into cDNA as previously described [27]. GAPDH was used to normalize gene expression levels in cells, and 28S rRNA was used as an internal control in teratoma analysis. Real-time quantitative reverse transcription PCR (qRT-PCR) analyses were performed on an ABI7900 using the FastStart Universal SYBR Green Master (Roche) as previously described [27]. Primers used for qRT-PCR analyses are listed in Additional file 1 (Table S1).

### Microarray assay and data analysis

Mouse ESC samples transfected with an empty vector or an Ssbp3 expression plasmid for 96 h were collected for gene expression detection using the Affymetrix GeneChip® Mouse Genome 430 2.0 Array. Duplicate samples were subjected to array analysis. The procedures including RNA extraction, cDNA synthesis, labeling, hybridization, washing, and scanning were performed by the Shanghai Biotechnology Corporation. Datasets were submitted to the database for annotation, visualization, and integrated discovery (DAVID), and analyzed by gene ontology (GO) analysis and Kyoto encyclopedia of genes and genomes (KEGG) pathway mapping. Data are publically available at the National Center for Biotechnology Information with Gene Expression Omnibus accession number GSE67562.

### Bisulfite sequencing

The bisulfite treatment of DNA was performed using the EZ-DNA methylation direct kit (Zymo Research) according to the manufacturer’s instructions. Elf5 primers were the same as described previously [28]. Amplified products were purified using gel filtration columns, cloned into the pGEM-T Easy Vector (Promega), and sequenced using SP6 primers. For each promoter sequence, ten randomly selected clones were sequenced.

### Teratomas

Mouse ESCs transfected with an empty vector or an Ssbp3 expression plasmid were selected with 1 μg/mL puromycin (Sigma) for 48 h. Then 1.5 × 10^6^ cells were intramuscularly injected into NOD/SCID mice [29]. Teratomas were collected 6 weeks later for qRT-PCR detection of gene expression and histological analysis.

### Chimera

Mouse ESCs constitutively expressing the H2B-GFP fusion gene were transfected with a vector or an Ssbp3 expression plasmid. Twenty-four hours later, cells were selected with 1 μg/mL puromycin for 2 days. Two cells were injected into each 8-cell-stage mouse embryo. The embryos were subsequently transferred into the uterus of pseudo-pregnant female mice following standard procedures. Injected embryos at embryonic day 6.5 (E6.5) and E14.5 were collected and imaged, respectively. Fluorescence images were taken by a laser confocal scanning microscope (Zeiss, LSM710) or a stereo zoom microscope (Zeiss, Axio Zoom.V16).

### Statistical analysis

All data were from three biological replicates and are presented as means ± SD. Pairwise statistical significance was determined by the Student’s *t* test*. p* < 0.05 was considered statistically significant.

## Results

### Forced expression of Ssbp3 induces differentiation of mouse ESCs with a trophoblast-like gene expression pattern

**Fig. 1:**
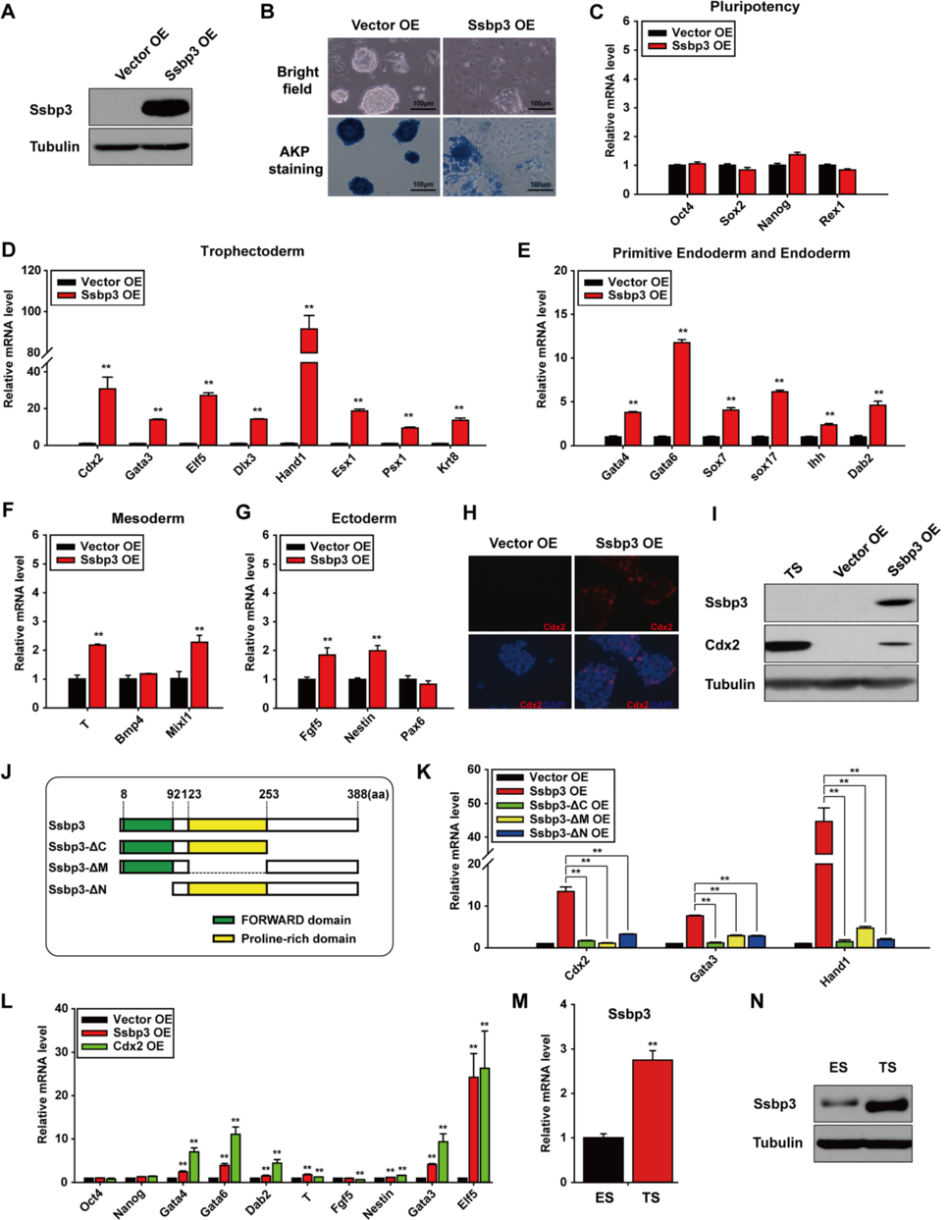
Forced expression of Ssbp3 induces differentiation of mouse ESCs with a trophoblast-like gene expression pattern. **a** Western blotting of Ssbp3 protein levels in ESCs transfected with a vector, or an Ssbp3 plasmid. Twenty-four hours after transfection, ESCs were selected by puromycin for an additional 72 h. **b** Morphology changes and AKP staining of ESCs overexpressing Ssbp3. **c**–**g** Expression levels of pluripotency and lineage markers in ESCs overexpressing Ssbp3 determined by qRT-PCR analyses. Pluripotency markers (**c**), trophoblast markers (**d**), primitive endoderm and endoderm markers (**e**), mesoderm markers (**f**), and ectoderm markers (**g**). The average mRNA level in cells transfected with the control vector was set at 1.0. Data are shown as mean ± SD (*n* = 3). *p < 0.05, **p < 0.01. **h** Immunofluorescence staining of ESCs 96 h after transfection. Samples were stained with anti-Cdx2 (*red*) antibody, and DAPI staining highlighted the nuclei (*blue*). **i** Western blotting of Cdx2 protein levels in ESCs 96 h after transfection. β-Tubulin was used as an internal control. **j** The schematic diagram of the Ssbp3 coding sequence and various truncation mutants. **k** qRT-PCR analysis for the expression levels of trophoblast markers in ESCs 96 h after transfection of plasmids as indicated. The average mRNA level in cells transfected with the control vector was set at 1.0. Data are shown as mean ± SD (*n* = 3). *p < 0.05, **p < 0.01. **l** qRT-PCR analysis for mRNA levels of pluripotency and lineage markers 96 h after transfection of indicated plasmids. The average mRNA level in cells transfected with the control vector was set at 1.0. Data are shown as mean ± SD (*n* = 3). *p < 0.05, **p < 0.01. **m**, **n** The mRNA and protein levels of Ssbp3 in ESCs and TSCs determined by qRT-PCR (**m**) and Western blotting (**n**). *ESC* embryonic stem cell, *OE* overexpression, *TSC* trophoblast stem cell

To study the function of Ssbp3 in mouse ESCs, we overexpressed Ssbp3 in E14T ESCs cultured under a self-renewal condition, in parallel with an empty vector as a negative control (Fig. 1a). Obvious differentiation was observed 96 h after transfection with the Ssbp3 plasmid, as evidenced by changes in the cell morphology and the intensity of alkaline phosphatase (AKP) staining (Fig. 1b). Our qRT-PCR results showed that the expression levels of key pluripotency-associated markers such as Oct4, Sox2, Nanog, and Rex1 were not altered significantly in ESCs overexpressing Ssbp3 compared with control ESCs at day 4 (Fig. 1c). However, most trophoblast marker genes tested, including early TSC markers (Cdx2, Gata3, Elf5) and relative mature trophoblast markers (Hand1, Dlx3, Esx1, Psx1, Krt8) [30], were significantly upregulated with greater than 10-fold increases (Fig. 1d), while most extra-embryonic endoderm marker genes detected such as Gata6, Gata4, Sox7, and Sox17 were modestly upregulated about five-fold (Fig. 1e). Meanwhile, expression levels of germ layer markers (the endoderm, mesoderm, and ectoderm) displayed mild alterations with less than two-fold changes (Fig. 1e, f and g). Consistently, immunofluorescence staining and Western blotting results revealed the enhanced expression of Cdx2 at the protein level 96 h after overexpression of Ssbp3 (Fig. 1h and i). We then evaluated the effect of Ssbp3 on ESCs under a differentiation condition induced by LIF withdrawal. Consistent with the results obtained under the self-renewal condition, trophoblast marker genes were dramatically upregulated (Figure S1A in Additional file 2). Overexpression of Ssbp3 also elevated extra-embryonic endoderm markers modestly (Figure S1B in Additional file 2) with slight alterations in the expression levels of germ layer-specific and pluripotency markers (Figure S1B, C, D and E in Additional file 2). These results implied that overexpression of Ssbp3 could induce mouse ESC differentiation biased towards trophoblast-like lineages.

Ssbp3 protein contains three different regions responsible for different functions: a well-conserved FORWARD/LUFS domain at the N-terminal end, through which Ssbp3 interacts with other proteins; a highly conserved proline-rich sequence in the middle critical for embryonic head development; and a C-terminal end possessing transcriptional activity [14, 31, 32]. To determine which region conferred Ssbp3 the ability to induce ESC differentiation, truncation mutants lacking the C-terminal, or middle, or N-terminal region were constructed (Fig. 1j) and transfected into ESCs, respectively. Unexpectedly, none of the truncation mutants displayed the same ESC differentiation function as did the full length Ssbp3 (Fig. 1k). Therefore, it is likely that the effect of Ssbp3 for inducing ESC differentiation requires its intact structure.

We next compared gene expression changes induced by Ssbp3 and Cdx2 overexpression, as Cdx2 is known as a key regulator for the trophoblast development, and overexpression of Cdx2 in mouse ESCs has been shown to efficiently induce trophoblast differentiation [7]. Our qRT-PCR results showed that expression patterns of various lineage markers in ESCs overexpressing Ssbp3 resembled those in ESCs overexpressing Cdx2 (Fig. 1l), suggesting that Ssbp3 might have a role similar to Cdx2 for induction of ESC differentiation. Moreover, we found that both mRNA and protein levels of Ssbp3 were substantially higher in TSCs than in ESCs, further supporting the association of Ssbp3 with trophoblast lineages at both mRNA and protein levels (Fig. 1m, n).

### Ssbp3 depletion attenuates the activation of trophoblast gene expression induced by downregulation of Oct4 in mouse ESCs

Mouse ESCs are usually considered to have a weak ability, if any, to generate trophoblast cell types by spontaneous differentiation [33]. However, genetic manipulation such as reduction of Oct4 or Tet1 [29, 34], or induction of Cdx2, Gata3, Arid3a, Brog5, or other key trophoblast-associated factors, can convert mouse ESCs to TS-like cells [6, 7, 35–38]. Here, we used the ZHBTc4 mouse ESC line as an in vitro differentiation model for trophoblast induction as previously reported [34]. In this cell line, both alleles of endogenous Oct4 loci were deleted and Oct4 expression was controlled by a tetracycline (Tc)-regulated Oct4 transgene.

**Fig. 2:**
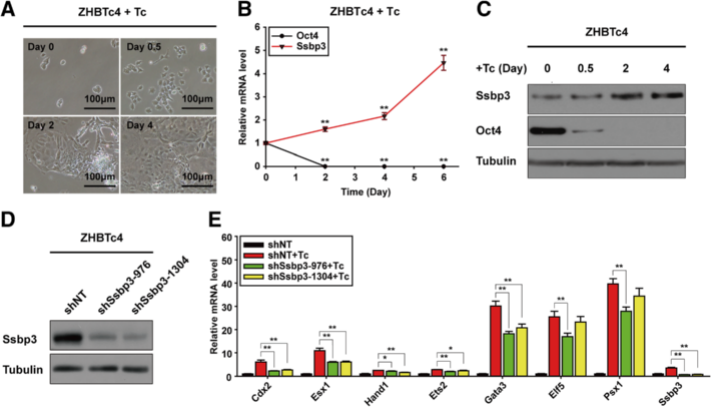
Ssbp3 depletion attenuates the activation of trophoblast gene expression induced by downregulation of Oct4 in mouse ESCs. **a** The morphology of ZHBTc4 cells after treatment with Tc. Differentiation was triggered by Tc-mediated downregulation of Oct4. **b** Expression levels of Ssbp3 during differentiation of the ZHBTc4 cell line were determined by qRT-PCR analysis. The average mRNA level in ZHBTc4 cells cultured without Tc was set at 1.0. Data are shown as mean ± SD (*n* = 3). *p < 0.05, **p < 0.01. **c** Protein levels of Ssbp3 and Oct4 during ZHBTc4 cell differentiation were determined by Western blotting. **d** Western blot analysis of the silencing efficiency of shRNAs targeting the Ssbp3 coding sequence in ZHBTc4 cells. **e** The expression levels of trophoblast-specific markers in cells expressing NT control shRNA or specific Ssbp3-targeting shRNA were determined by qRT-PCR analysis. Expression of Oct4 was downregulated by treatment with Tc for 96 h in ZHBTc4 ESCs. The average mRNA level in shNT-expressing ZHBTc4 cells cultured in the absence of Tc was set at 1.0. Data are shown as mean ± SD (*n* = 3). *p < 0.05, **p < 0.01. *NT* non-targeting, *shRNA* short-hairpin RNA, *Tc* tetracycline

In line with published results, Tc treatment reduced Oct4 expression rapidly at both the mRNA and protein levels, and robustly induced trophoblast differentiation (Fig. 2a, b, c). We found that the expression of Ssbp3 at the mRNA and protein levels increased gradually with Tc treatment (Fig. 2b, c), adding more evidence for the potential association of Ssbp3 expression with trophoblast differentiation.

To determine whether Ssbp3 plays a role in the trophoblast differentiation induced by Oct4 downregulation, two shRNA sequences (shSsbp3-976 and shSsbp3-1304) targeting different Ssbp3 coding sequences were used to establish two stable Ssbp3 knockdown ZHBTc4 ESC lines. A control cell line (shNT) was generated using a non-targeting shRNA sequence (Fig. 2d). qRT-PCR results showed that Tc treatment for 96 h induced expression of trophoblast lineage markers in shNT cells as reported previously [34]. However, levels of these trophoblast markers were markedly lower in shSsbp3-976 and shSsbp3-1304 cells than in control cells, although they were still higher than those in ESCs in the absence of Tc treatment (Fig. 2e). Hence, Ssbp3 was partially required for activation of trophoblast marker genes induced by Oct4 depletion.

### Ssbp3 depletion weakens the trophoblast gene expression induced by BMP4 and bFGF treatment in ESCs

**Fig. 3:**
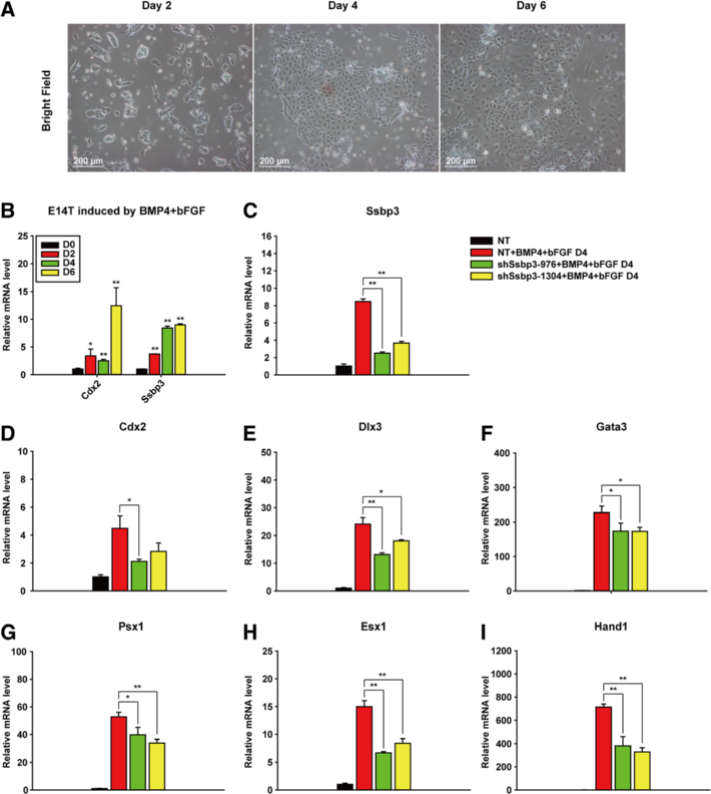
Ssbp3 depletion weakens the trophoblast gene expression induced by BMP4 and bFGF treatment in ESCs. **a** The morphology of E14T cells after treatment with bone BMP4 and bFGF at the indicated time points. **b** Expression levels of Ssbp3 gradually increased in E14T cells treated with BMP4 and bFGF. The expression levels of Ssbp3 were determined by qRT-PCR analysis. The average mRNA level in E14T cells cultured without treatment was set at 1.0. Data are shown as mean ± SD (*n* = 3). *p < 0.05, **p < 0.01. **c** The silencing efficiency of shRNAs targeting Ssbp3 in E14T cells was detected by qRT-PCR analysis at day 4. **d**–**i** The expression levels of trophoblast-specific markers in cells expressing NT control shRNA or specific Ssbp3-targeting shRNAs were determined by qRT-PCR analysis. E14T cells were induced to trophoblast differentiation by treatment with BMP4 and bFGF for 96 h. The average mRNA level in shNT-expressing E14T cells without treatment was set at 1.0. Data are shown as mean ± SD (*n* = 3). *p < 0.05, **p < 0.01. *bFGF* basic fibroblast growth factor, *BMP4* bone morphogenetic protein 4, *NT* non-targeting, *shRNA* short-hairpin RNA

To determine the role of Ssbp3 in an additional trophoblast differentiation model, we developed a new cytokine-induced trophoblast differentiation protocol based on two published protocols. One protocol was developed in Hubert Schorle’s laboratory using a chemically defined TX medium containing bFGF and TGF-β [23]; the other protocol used BMP4 to enable mouse ESCs to differentiate into trophoblasts in defined culture conditions [39]. In this study, we used BMP4 to replace TGF-β in the TX medium and named the new medium the Bb medium, as we found that the combination of BMP4 and bFGF in the TX medium could model ESC differentiation into trophoblast lineages more efficiently. Cells lost typical ESC morphology and became flat 2 days after they were cultured in the Bb medium. The majority of the cells became cobblestone-shaped at day 6 (Fig. 3a). Transcript levels of Cdx2 and Ssbp3 increased gradually during the differentiation (Fig. 3b).

To investigate the role of Ssbp3 in this triggering agent-induced trophoblast differentiation model, we established two Ssbp3 stable knockdown E14T lines using the same shRNA sequences mentioned above (Fig. 3c); a control line was generated with the shNT sequence. At day 4 of the Bb medium-induced differentiation, the expression of trophoblast transcription factors (Dlx3, Gata3, Psx1, Esx1, and Hand1) were evidently upregulated, with elevated levels ranging from 15-fold to several hundred-fold compared with levels in undifferentiated ESCs (Fig. 3e–i), further validating that the newly developed protocol could robustly induce trophoblast differentiation in ESCs. In accordance with the results obtained in the Oct4 reduction-induced trophoblast differentiation model, depletion of Ssbp3 attenuated the induction of most trophoblast markers significantly (Fig. 3d–i), supporting the hypothesis that Ssbp3 plays an important role for the full induction of trophoblast lineage markers during ESC differentiation into trophoblast-like cells.

### Overexpression of Ssbp3 induces a trophoblast-like transcriptional program

**Fig. 4:**
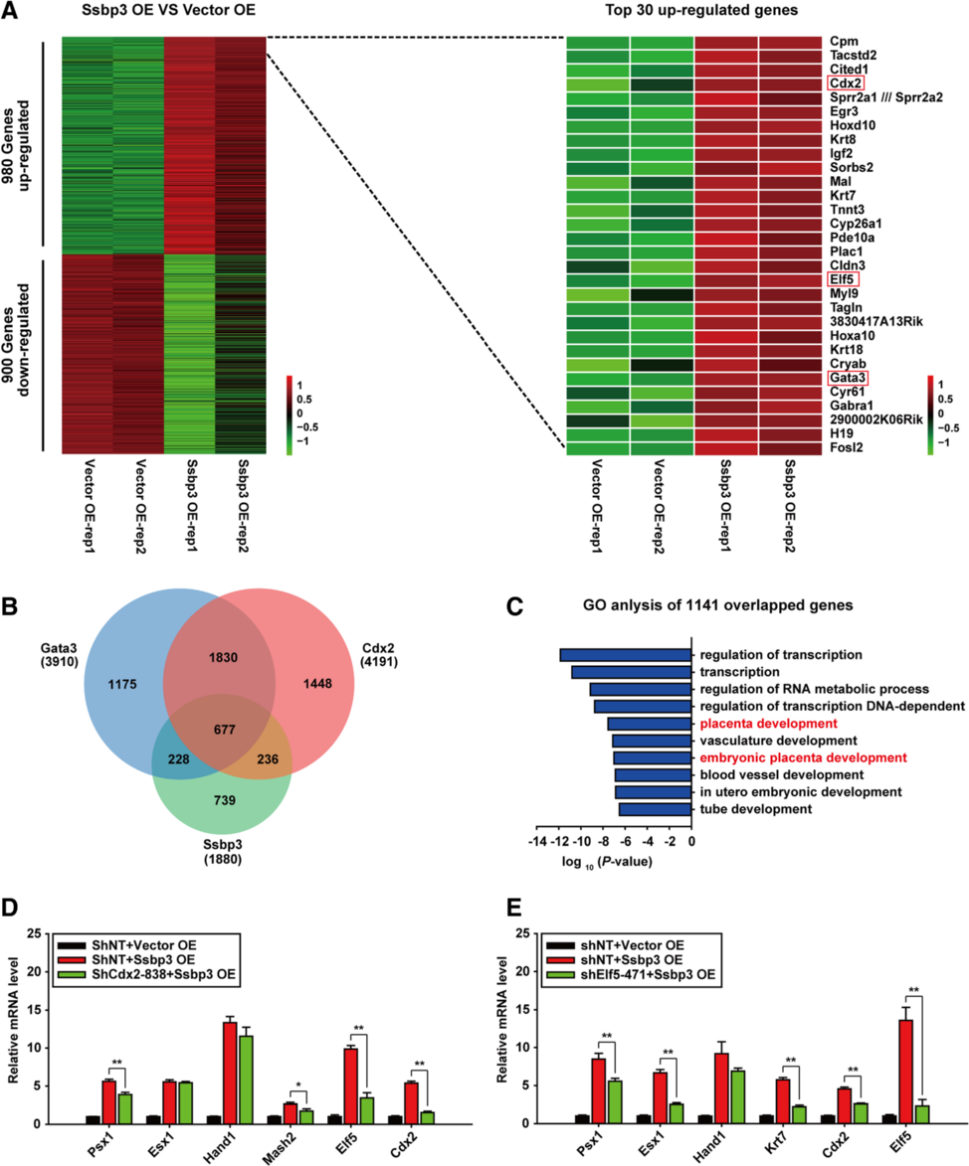
Overexpression of Ssbp3 induces a trophoblast-like transcriptional program. **a** Heatmap of the DEGs induced by Ssbp3 overexpression in ESCs (fold change >2). *Green* and *red* values represent fold changes for down- and upregulation, respectively. Heatmap in the *right panel* shows the top 30 upregulated genes in detail. **b** Venn diagram showing the overlap of the DEGs induced by Ssbp3 (*green*), Gata3 (*blue*), or Cdx2 (*orange*) overexpression, with the number of genes indicated. Out of 1880 DEGs induced by Ssbp3, 1141 DEGs were shared with Gata3 or Cdx2. **c** Significantly enriched GO terms of the 1141 DEGs shared between Ssbp3 and Cdx2 or between Ssbp3 and Gata3. **d**, **e** qRT-PCR analysis for expression levels of trophoblast-specific markers in Cdx2 and Elf5 stable knockdown cell lines 96 h after Ssbp3 overexpression. The average mRNA level in stable cell line expressing shNT was set at 1.0. Data are shown as mean ± SD (*n* = 3). *p < 0.05, **p < 0.01. *GO* gene ontology, *NT* non-targeting, *OE* overexpression, *shRNA * short-hairpin RNA

To investigate the transcriptional effect of ectopic expression of Ssbp3 on a genome-wide scale, we compared the global transcriptional profile of Ssbp3-overexpressing cells with that of control cells using affymetrix microarray. Biological duplicate samples were used in this assay. One thousand eight hundred and eighty differentially expressed genes (DEG) were identified with a cutoff threshold of two-fold, including 980 upregulated and 900 downregulated genes, respectively (Fig. 4a, left panel; Table S2 in Additional file 3). The top 30 DEGs induced were listed in the heatmap shown in the right panel of Fig. 4a. As anticipated, the majority of the listed top genes are specifically related to the trophoblast development. Notably, critical TSC master regulators such as Cdx2, Elf5 and Gata3 were included in the list. The findings suggested that ectopic expression of Ssbp3 evoked an overall startup of a trophoblast-like transcriptional program in mouse ESCs.

To further define the ability of Ssbp3 to induce ESC differentiation to a trophoblast-like phenotype, we compared the global expression profile of Ssbp3-overexpressing ESCs with previously published profiles of Cdx2- and Gata3-overexpressing ESCs [6]. As shown, 60.1 % (1141 out of the 1880) of the DEGs induced by Ssbp3 were shared with the DEGs induced by Cdx2 or Gata3 (Fig. 4b; Table S3 in Additional file 4). GO analysis illustrated that these overlapped genes were strongly enriched in terms associated with transcription regulation and placenta development (Fig. 4c). Moreover, we analyzed the upregulated genes in Ssbp3-overexpressing cells using a batch query tool at the website of the Mouse Genome Informatics (MGI). Recovered mammalian phenotype (MP) terms contained multiple trophoblast subtypes and developmental stages (Table S4 in Additional file 5), similar to the MP terms recovered in Cdx2- and Gata3-overexpressing cells [6]. These results further validated the role of Ssbp3 for inducing a trophoblast-like transcriptional program in mouse ESCs. In addition, the remaining 39.9 % (739 out of the 1880) of the DEGs specifically induced by Ssbp3 (Fig. 4b; Table S3 in Additional file 4) were also analyzed by GO analysis, and they were most enriched in terms related to skeletal system development and morphogenesis (Figure S2 in Additional file 6), well in accordance with the previously reported phenotype of truncation of anterior skull bones and mild skeletal defects in other body parts in Ssbp3 knockout mice [13, 14].

Since TS-specific master genes Cdx2 and Elf5 were listed among the top 20 upregulated genes induced by overexpression of Ssbp3, we were interested to know whether Cdx2 or Elf5 acted as the downstream factors of Ssbp3, and were therefore involved in the trophoblast-like differentiation program from ESCs driven by exogenous expression of Ssbp3. To address this question, shRNAs based Cdx2 or Elf5 stable knockdown ESC lines were established. The results of qRT-PCR analyses revealed that knockdown of either Cdx2 or Elf5 obviously compromised the ability of Ssbp3 to induce the expression of trophoblast marker genes (Fig. 4d, e), indicating that Cdx2 and Elf5 probably play important roles for Ssbp3 to drive the trophoblast-like transcription program from ESCs.

### Overexpression of Ssbp3 decreases the methylation level at the Elf5 promoter in mouse ESCs

**Fig. 5:**
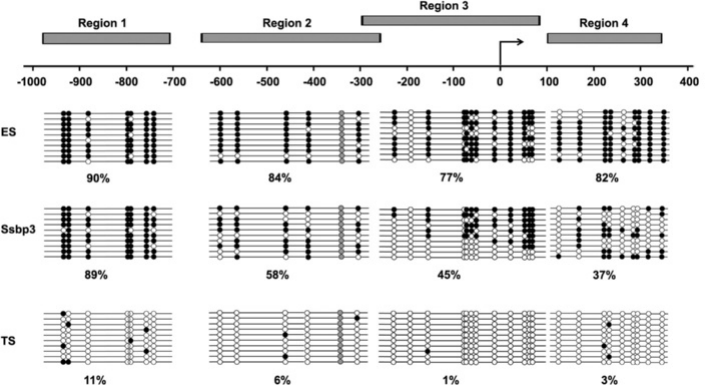
Overexpression of Ssbp3 decreases the methylation level at the Elf5 promoter in mouse ESCs. Clonal bisulfite sequencing of the 1 kb promoter region of Elf5 in ESCs, TSCs, and ESCs overexpressing Ssbp3 were shown. Each line represents an individual clone with *open circles* depicting unmethylated sites and *filled circles* indicating methylated sites. The CpG dinucleotide at –355 bp is polymorphic and absent where shaded *gray. ES* *C* embryonic stem cell, *TSC* trophoblast stem cell

The methylation status of the Elf5 promoter was reported to have a robust difference between ESCs and TSCs [40, 41], with a methylated and repressive status in ESCs and a hypo-methylated and active status in TSCs. We compared the methylation status at four separate regions of the Elf5 promoter positioning from –1000 bp to +400 bp with respect to the transcription start site (TSS) among ESCs overexpressing Ssbp3 at day 6, undifferentiated ESCs, and TSCs. Bisulfite sequencing analysis showed that all regions were extensively methylated in undifferentiated ESCs but hypo-methylated in TSCs. However, in Ssbp3-overexpressing ESCs, the percentage of methylated CpG dinucleotides was higher in all four regions compared to TSCs, but lower in regions 2, 3 and 4 compared to undifferentiated ESCs (Fig. 5). Thus, it seems that overexpression of Ssbp3 in ESCs under a self-renewal culture condition generated a specific Elf5 promoter methylation pattern in which the methylation level was between undifferentiated ESCs and TSCs, in line with the elevated Elf5 mRNA level in Ssbp3-overexpressing ESCs.

### Ssbp3 overexpression activates MAPK/Erk1/2 and TGF-β pathways

**Fig. 6:**
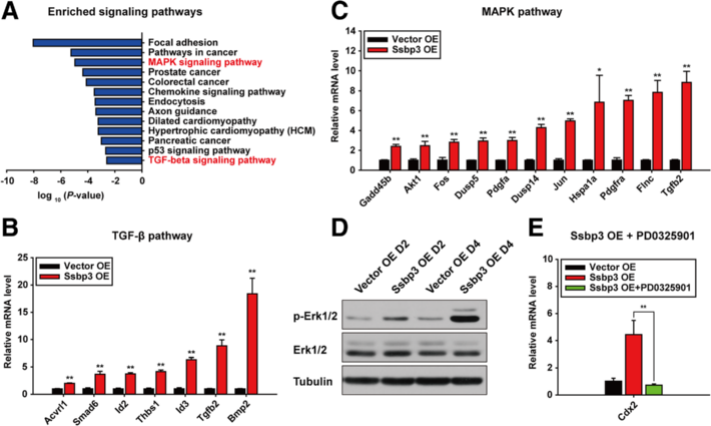
Ssbp3 overexpression activates MAPK/Erk1/2 and TGF-β pathways. **a** Significantly enriched signaling pathways of all DEGs upon overexpression of Ssbp3 by KEGG pathway analysis. **b**, **c** qRT-PCR analysis for expression levels of upregulated genes related to MAPK/Erk/1/2 and TGF-β pathways in Ssbp3-overexpressing ESCs. The average mRNA level in cells transfected with the control vector was set at 1.0. Data are shown as mean ± SD (*n* = 3). *p < 0.05, **p < 0.01. **d** Western blotting of p-Erk1/2 and total Erk1/2 protein levels in Ssbp3-overexpressing cells. Cells transfected with an Ssbp3 plasmid or a vector were collected at 48 h and 96 h after transfection, respectively. **e** qRT-PCR analysis for expression levels of Cdx2 in Ssbp3-overexpressing ESCs with or without PD0325901 treatment. The average mRNA level in cells transfected with the control vector in the absence of PD0325901 was set at 1.0. Data are shown as mean ± SD (*n* = 3). *p < 0.05, **p < 0.01. *Erk* extracellular signal-regulated kinase, *MAPK* mitogen-activated protein kinase, *OE* overexpression, *TGF* transforming growth factor

Trophoblast differentiation involves multiple signaling pathways. We examined which pathway was activated upon Ssbp3 overexpression. KEGG pathway analysis of all 1880 DEGs induced by Ssbp3 overexpression showed that MAPK/Erk1/2 signaling and TGF-β signaling pathways were markedly enriched (Fig. 6a). These two pathways had been previously reported to be critical for establishment of the trophectoderm in vivo or maintenance of TSC proliferation in vitro [18, 20]. Our qRT-PCR analysis for these signaling-related components verified their activation in Ssbp3-overexpressing cells (Fig. 6b, c). Besides, Western blotting showed enhanced phosphorylation levels of Erk1/2 in Ssbp3-overexpressing cells, suggesting a possible interplay between Ssbp3 and the MAPK/Erk1/2 pathway (Fig. 6d). Functionally, treatment of Ssbp3-overexpressing ESCs with a MAP kinase-ERK kinase (MEK) inhibitor, PD0325901 [42], diminished the upregulation of Cdx2 induced by Ssbp3 overexpression (Fig. 6e). The result was consistent with the report that PD0325901 treatment impaired Cdx2 expression and function in early embryos [18]. Taken together, these data indicated that Ssbp3 could activate MAPK/Erk1/2 and TGF-β pathways, which might be, at least partially, responsible for the activation of the trophoblast lineage marker genes induced by Ssbp3 overexpression.

### Teratomas derived from Ssbp3-overexpressing ESCs contain hemorrhage

**Fig. 7:**
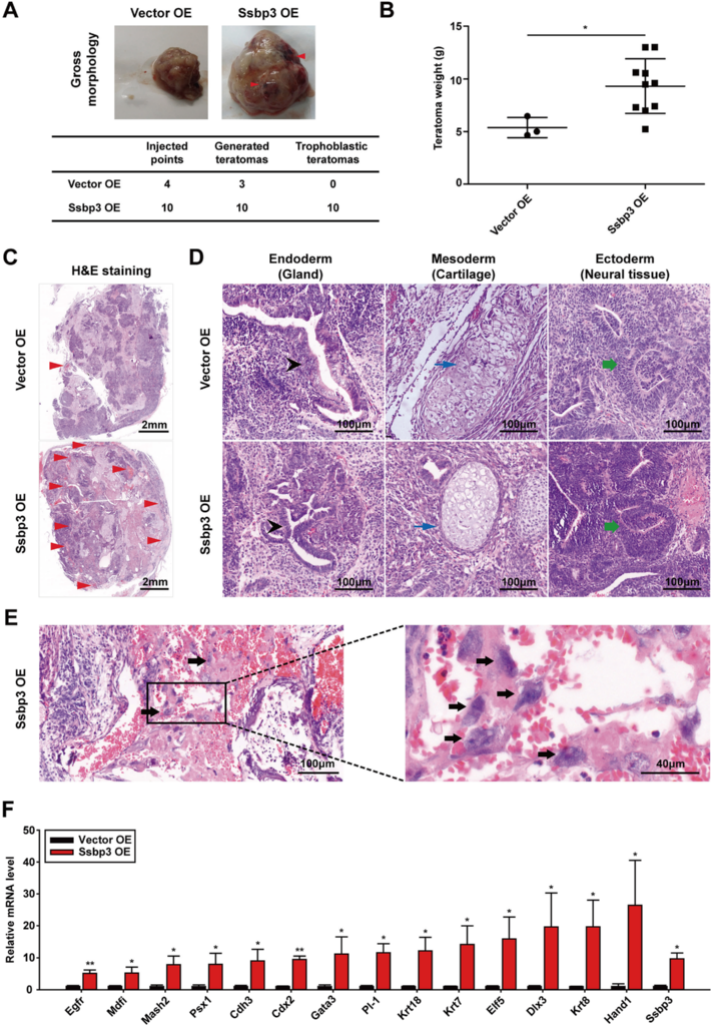
Teratomas derived from Ssbp3-overexpressing ESCs contain hemorrhage. **a** Gross appearance of teratomas derived from control cells or Ssbp3-overexpressing ESCs (*upper panel*). The number of teratomas examined is presented in the table (*lower panel*). **b** The net weight of teratomas derived from control cells and Ssbp3-overexpressing ESCs. **c** Cross-section of teratomas derived from control cells (*upper panel*) or Ssbp3-overexpressing ESCs (*lower panel*). **d** Histology of teratomas derived from control cells (*upper panel*) or Ssbp3-overexpressing ESCs (*lower panel*) showing tissue complexity (hematoxylin and eosin staining). *Arrowheads* mark the endoderm (*black*), mesoderm (*blue*), and ectoderm (*green*) cells. **e** Hematoxylin and eosin-stained images for sections of a teratoma derived from Ssbp3-overexpressing ESCs. The trophoblast cluster (*arrow heads*, *left panel*) and trophoblast giant cells with the enlarged nuclei (*arrow heads*, *right panel*) are indicated. **f** qRT-PCR analysis for expression levels of trophoblast-specific markers in teratomas derived from control cells or Ssbp3-overexpressing ESCs. The average mRNA level in teratomas derived from control cells was set at 1.0. Data are shown as mean ± SD (*n* = 3). *p < 0.05, **p < 0.01. *OE* overexpression

To test the developmental potential of Ssbp3-overexpressing ESCs in vivo, ESCs transfected with the Ssbp3 expression plasmid were intramuscularly injected into NOD/SCID mice. ESCs transfected with an empty vector were injected as a negative control. Teratomas were obtained from these mice 6 weeks later. Three teratomas were generated from vector-transfected ESCs and 10 teratomas were generated from Ssbp3-overexpressing ESCs (Fig. 7a). Teratomas generated from Ssbp3-overexpressing ESCs were larger and heavier compared with teratomas derived from control ESCs (Fig. 7a and b). Of note, Ssbp3-overexpressing ESC-produced teratomas contained obvious hemorrhage. Histologically, both types of teratomas contained tissues and cells representing all three embryonic germ layers (Fig. 7c and d). However, clusters of trophoblast giant cells possessing large cytoplasm and large nuclei could be apparently observed in the internal hemorrhagic regions of Ssbp3-overexpressing ESC-produced teratomas, but not in control ESC-generated teratomas (Fig. 7e). Furthermore, expression levels of trophoblast markers were much higher in teratomas derived from Ssbp3-overexpressing ESCs compared with control teratomas (Fig. 7f). Therefore, overexpression of Ssbp3 aroused the tendency of ESCs to differentiate into trophoblast-like cell types in vivo.

### ESCs overexpressing Ssbp3 mainly contribute to the placenta part in chimeric embryos

To study the in vivo differentiation ability of ESCs overexpressing Ssbp3, we injected mouse ESCs genetically labeled with green fluorescent protein (GFP) and overexpressing Ssbp3 into 8-cell-stage embryos (E2.5) and traced their distribution at E6.5 and E14.5, respectively (Figure S3A in Additional file 7). We carried out two batches of experiments. In the first batch of experiment, we injected 30 embryos with control cells overexpressing a vector and 50 embryos with ESCs overexpressing Ssbp3. At E6.5, one embryo injected with the control ESCs and nine embryos injected with ESCs overexpressing Ssbp3 were obtained. In contrast to the whole embryo distribution of vector-transfected ESCs, in six out of nine embryos, ESCs overexpressing Ssbp3 were located predominantly in the trophoblast giant cell region and ectoplacental cone, as well as in extra-embryonic endoderm, but not in the epiblast of E6.5 embryos (Figure S3B in Additional file 7). In the second batch of experiments, we injected 50 embryos with control cells and 50 embryos with Ssbp3-overexpressing ESCs. At E14.5, out of 100 injected embryos, only one embryo injected with Ssbp3-overexpressing ESCs was obtained. Consistent with the result obtained in the first batch of experiments, the Ssbp3-overexpressing ESCs mainly contributed to the placenta part (Figure S3C in Additional file 7). These data suggest an active role of Ssbp3 for promoting trophoblast-like lineage differentiation in vivo.

## Discussion

The present study shows that Ssbp3 might be an important regulator of trophoblast lineage differentiation. The following lines of experimental evidence support this proposal: i) Ssbp3 was highly expressed in TSCs, and its expression gradually increased during ESC trophoblast differentiation induced by Oct4 knockdown or treatment with BMP4 and bFGF; ii) overexpression of Ssbp3 dramatically induced the expression of trophoblast marker genes; iii) depletion of Ssbp3 attenuated the induction of trophoblast-associated genes induced by downregulation of Oct4 or treatment with BMP4 and bFGF in ESCs; iv) Ssbp3-overexpressing ESCs generated large aggressive teratomas with massive internal hemorrhage in vivo; and v) when injected into 8-cell-stage embryos, Ssbp3-overexpressing ESCs mainly contributed to the placenta part. Interestingly, downregulation of Ssbp3 decreased the induction of Cdx2 and Esx1 more dramatically than other trophoblast marker genes when Oct4 was downregulated or when BMP4 and bFGF were included in the ESC culture medium. It is possible that Ssbp3, as a DNA binding protein, regulates Cdx2 and Esx1 directly, although we do not have any experimental evidence for this.

Our global gene expression analysis revealed that Ssbp3 could drive a trophoblast-like transcriptional program in ESCs. About 60 % of the 1880 DEGs induced by Ssbp3 overexpression overlapped with the previously reported DEGs induced by Cdx2 or Gata3 [6], indicating that these three regulators may share functional mechanisms to drive trophoblast differentiation. GO analysis illustrated that the DEGs shared by Ssbp3 and Cdx2 or Ssbp3 and Gata3 were highly enriched in terms associated with transcription regulation and placenta development. Cdx2 and Elf5, two key regulators of TSC self-renewal and maintenance, were robustly induced by ectopic expression of Ssbp3. Notably, Cdx2 or Elf5 depletion impaired activation of trophoblast lineage marker genes induced by Ssbp3 overexpression. These findings imply that Ssbp3 may act as an upstream regulator for the expression of Cdx2 and Elf5 during trophoblast differentiation, although it is not clear whether Ssbp3 executes this function directly or not. Although Ssbp3, Cdx2, and Gata3 can all activate trophoblast lineage marker genes, each has its distinct targets. In fact, GO analysis demonstrates that the specific DEGs regulated by Ssbp3 are strongly related to embryonic skeletal system development and the pattern specification process, indicating that Ssbp3 plays a role in embryogenesis as previously reported [13, 14]. Furthermore, KEGG analysis showed that MAPK/Erk1/2 and TGF-β pathways were activated in Ssbp3-overexpressing ESCs. All of the bioinformatic analyses, including GO, KEGG, and MP, support the hypothesis that Ssbp3 is closely associated with the transcriptional regulatory circuitry of trophoblast cell differentiation. As Ssbp3 was highly expressed in TSCs and activated transcription factors and signaling pathways required for TSC self-renewal, we propose that Ssbp3 may function at, or from, an early stage of trophoblast lineage specification.

The detailed mechanism by which Ssbp3 executed its function in regulation of trophoblast gene expression is not clear yet. Based on the facts that the overlapped DEGs between Ssbp3 and Cdx2 or Ssbp3 and Gata3 were strongly enriched in terms associated with transcriptional regulation and that Ssbp3 was reported to have transcriptional activity in its C-terminus [32], we anticipate that it may function through regulating gene expression as a transcription factor. However, where and how it binds the genome remains unclear. In fact, Ssbp3 binding sites, including both single- and double-stranded DNAs, are still rarely identified. So far only a pyrimidine-rich element in the promoter region of the chicken α2 (I) collagen gene was identified as a single-stranded DNA binding site by Ssbp3 [10]. Further investigations with chromatin immunoprecipitation coupled with DNA-sequencing (ChIP-seq) assays may help to address this question.

Our study shows that Ssbp3 plays an important role during trophoblast lineage specification in vitro. However, previous studies reported that Ssbp3 null mice showed skeletal abnormalities, but nothing was mentioned about placental abnormalities or embryonic lethality [11, 12]. The lack of early embryonic lethality (at or before implantation) in Ssbp3 knockout mice might be explained by functional redundancy from other family members. Supporting this hypothesis, we found that Ssbp2, another member of the Ssbp3 family, had a similar role to Ssbp3. Overexpression of Ssbp2 changed ESC morphology and led to increased expression of trophoblast-associated markers to an extent more dramatic than other lineage markers (data not shown). Therefore, double-knockout of Ssbp3 and Ssbp2 in mice could be helpful to verify the assumption.

It is known that Ssbp3 plays a critical role in the head morphogenesis during mouse embryonic development through activating the Lim1-Ldb1 transcriptional complex via interaction with Ldb1 [13]. To explore the effect of Ldb1 on the induction of trophoblast marker gene expression induced by Ssbp3, we up- and downregulated Ldb1 in ESCs together with Ssbp3 overexpression, respectively. No obvious effects were found (data not shown), suggesting that the trophoblastic gene induction activity of Ssbp3 may be Ldb1-independent. Further exploration of proteins interacting with Ssbp3 will facilitate our understanding of how Ssbp3 controls the trophoblast-like transcriptional program and activates related pathways.

## Conclusions

Ectopic expression of Ssbp3 robustly induced trophoblast lineage marker gene expression in mouse ESCs. Conversely, depletion of Ssbp3 attenuated the trophoblast gene activation. Ssbp3 controlled a trophoblast-like transcriptional program by inducing the expression of early trophectoderm master transcription factors such as Cdx2, Gata3, and Elf5, and by activating MAPK/Erk1/2 and TGF-β pathways, two signaling pathways essential for trophoblast development. Both gain- and loss-of-function experiments support the notion that Ssbp3 might be an important regulator involved in trophoblast differentiation from mouse ESCs. The study is significant for better understanding the regulatory networks controlling ESC differentiation into extra-embryonic lineages and should shed light on the study of trophoblast development of mouse early embryos.

## Abbreviations

AKP, alkaline phosphatase; bFGF, basic fibroblast growth factor; BMP, bone morphogenetic protein; BSA, bovine serum albumin; cDNA, complementary DNA; DAPI, 4',6-diamidino-2-phenylindole; DAVID, database for annotation, visualization, and integrated discovery; DEG, differentially expressed genes; DMEM, Dulbecco's modified Eagle’s medium; E, embryonic day; ERK, extracellular signal-regulated kinase; ESC, embryonic stem cell; FBS, fetal bovine serum; FGF4, fibroblast growth factor 4; GO, gene ontology; ICM, inner cell mass; KEGG, Kyoto encyclopedia of genes and genomes; LIF, leukemia inhibitory factor; MAPK, mitogen-activated protein kinase; MEF, mouse embryonic fibroblast; MEK, MAP kinase-ERK kinase; MGI, Mouse Genome Informatics; MP, mammalian phenotype; PCR, polymerase chain reaction; qRT-PCR, real-time quantitative reverse transcription polymerase chain reaction; shRNA, short-hairpin RNA; Tc, tetracycline; TE, trophectoderm; TGF, transforming growth factor; TSC, trophoblast stem cell

## Additional files

Additional file 1: Table S1. Primers used in this study for gene cloning, qRT-PCR, and shRNA targeting sequences. (XLSX 16 kb) Additional file 2: Figure S1. Forced expression of Ssbp3 induces mouse ESC differentiation with a bias to trophoblast lineages under the LIF withdrawal condition. (A–E) Expression levels of pluripotency and lineage specific markers in ESCs overexpressing Ssbp3 were determined by qRT-PCR analyses, including markers for the trophoblast (A), primitive and definitive endoderm (B), mesoderm (C), ectoderm (D), and pluripotency (E). The average mRNA level in cells transfected with the control vector was set at 1.0. Data are shown as mean ± SD (*n* = 3). **p* < 0.05, ***p* < 0.01. (TIF 306 kb) Additional file 3: Table S2. Differentially expressed genes induced by Ssbp3. (XLSX 36 kb) Additional file 4: Table S3. Shared or specific DEGs induced by Ssbp3, Cdx2, or Gata3 overexpression. (XLSX 110 kb) Additional file 5: Table S4. MGI Batch Report—mammalian phenotype (MP) ontology of the upregulated genes induced by Ssbp3 overexpression. (XLSX 15 kb) Additional file 6: Figure S2. GO analysis of 739 Ssbp3 specific genes compared with DEGs induced by Gata3 or Cdx2 overexpression. The significantly enriched GO terms are strongly related to embryonic skeletal system development and pattern specification process. (TIF 78 kb) Additional file 7: Figure S3. Ssbp3-overexpressing ESCs mainly contribute to the placenta part in chimeric embryos. (A) GFP-labeled E14T cells overexpressing a vector or an Ssbp3 expression plasmid were injected into wild-type 8-cell-stage embryos. For each embryo, two GFP-labeled cells were injected. (B) Strong contribution of injected E14T cells overexpressing Ssbp3 to the extra-embryonic lineage in E6.5 chimeric embryos. Gross and fluorescence images of E6.5 chimeric embryos developed from embryos described in A. (C) E14T cells overexpressing Ssbp3 predominantly contributed to the placenta part of the E14.5 chimeric embryo. (TIF 7678 kb)

## References

[1] Lanner F (2014). Lineage specification in the early mouse embryo. Exp Cell Res.

[2] Cockburn K, Rossant J (2010). Making the blastocyst: lessons from the mouse. J Clin Invest.

[3] Fleming TP (1987). A quantitative analysis of cell allocation to trophectoderm and inner cell mass in the mouse blastocyst. Dev Biol.

[4] Evans MJ, Kaufman MH (1981). Establishment in culture of pluripotential cells from mouse embryos. Nature.

[5] Tanaka S, Kunath T, Hadjantonakis AK, Nagy A, Rossant J (1998). Promotion of trophoblast stem cell proliferation by FGF4. Science.

[6] Ralston A, Cox BJ, Nishioka N, Sasaki H, Chea E, Rugg-Gunn P, Guo G, Robson P, Draper JS, Rossant J. Gata3 regulates trophoblast development downstream of Tead4 and in parallel to Cdx2. Development. 2010;137:395–403. 10.1242/dev.038828 20081188

[7] Tolkunova E, Cavaleri F, Eckardt S, Reinbold R, Christenson LK, Scholer HR, Tomilin A. The caudal-related protein cdx2 promotes trophoblast differentiation of mouse embryonic stem cells. Stem Cells. 2006;24:139–44. 10.1634/stemcells.2005-0240 16210407

[8] Home P, Ray S, Dutta D, Bronshteyn I, Larson M, Paul S (2009). GATA3 is selectively expressed in the trophectoderm of peri-implantation embryo and directly regulates Cdx2 gene expression. J Biol Chem.

[9] Castro P, Liang H, Liang JC, Nagarajan L (2002). A novel, evolutionarily conserved gene family with putative sequence-specific single-stranded DNA-binding activity. Genomics.

[10] Bayarsaihan D, Soto RJ, Lukens LN (1998). Cloning and characterization of a novel sequence-specific single-stranded-DNA-binding protein. Biochem J.

[11] Chen L, Segal D, Hukriede NA, Podtelejnikov AV, Bayarsaihan D, Kennison JA, Ogryzko VV, Dawid IB, Westphal H. Ssdp proteins interact with the LIM-domain-binding protein Ldb1 to regulate development. Proc Natl Acad Sci U S A. 2002;99:14320–5. 10.1073/pnas.212532399 PMC137882 12381786

[12] van Meyel DJ, Thomas JB, Agulnick AD. Ssdp proteins bind to LIM-interacting co-factors and regulate the activity of LIM-homeodomain protein complexes in vivo. Development. 2003;130:1915–25. 10.1242/dev.00389 12642495

[13] Nishioka N, Nagano S, Nakayama R, Kiyonari H, Ijiri T, Taniguchi K, Shawlot W, Hayashizaki Y, Westphal H, Behringer RR, Matsuda Y, Sakoda S, Kondoh H, Sasaki H. Ssdp1 regulates head morphogenesis of mouse embryos by activating the Lim1-Ldb1 complex. Development. 2005;132:2535–46. 10.1242/dev.01844 15857913

[14] Enkhmandakh B, Makeyev AV, Bayarsaihan D (2006). The role of the proline-rich domain of Ssdp1 in the modular architecture of the vertebrate head organizer. Proc Natl Acad Sci U S A.

[15] Gungor C, Taniguchi-Ishigaki N, Ma H, Drung A, Tursun B, Ostendorff HP, Bossenz M, Becker CG, Becker T, Bach I. Proteasomal selection of multiprotein complexes recruited by LIM homeodomain transcription factors. Proc Natl Acad Sci U S A. 2007;104:15000–5. 10.1073/pnas.0703738104 PMC1986602 17848518

[16] Xu Z, Meng X, Cai Y, Liang H, Nagarajan L, Brandt SJ (2007). Single-stranded DNA-binding proteins regulate the abundance of LIM domain and LIM domain-binding proteins. Genes Dev.

[17] Saba-El-Leil MK, Vella FD, Vernay B, Voisin L, Chen L, Labrecque N, Ang SL, Meloche S. An essential function of the mitogen-activated protein kinase Erk2 in mouse trophoblast development. EMBO Rep. 2003;4:964–8. 10.1038/sj.embor.embor939 PMC1326397 14502223

[18] Lu CW, Yabuuchi A, Chen L, Viswanathan S, Kim K, Daley GQ (2008). Ras-MAPK signaling promotes trophectoderm formation from embryonic stem cells and mouse embryos. Nat Genet.

[19] Slager HG, Lawson KA, van den Eijnden-van Raaij AJ, de Laat SW, Mummery CL (1991). Differential localization of TGF-beta 2 in mouse preimplantation and early postimplantation development. Dev Biol.

[20] Erlebacher A, Price KA, Glimcher LH (2004). Maintenance of mouse trophoblast stem cell proliferation by TGF-beta/activin. Dev Biol.

[21] Quinn J, Kunath T, Rossant J (2006). Mouse trophoblast stem cells. Methods Mol Med.

[22] Lu R, Yang A, Jin Y (2011). Dual functions of T-box 3 (Tbx3) in the control of self-renewal and extraembryonic endoderm differentiation in mouse embryonic stem cells. J Biol Chem.

[23] Kubaczka C, Senner C, Arauzo-Bravo MJ, Sharma N, Kuckenberg P, Becker A, Zimmer A, Brustle O, Peitz M, Hemberger M, Schorle H. Derivation and maintenance of murine trophoblast stem cells under defined conditions. Stem Cell Rep. 2014;2:232–42. 10.1016/j.stemcr.2013.12.013 PMC3923226 24527396

[24] Stewart SA, Dykxhoorn DM, Palliser D, Mizuno H, Yu EY, An DS, Sabatini DM, Chen IS, Hahn WC, Sharp PA, Weinberg RA, Novina CD. Lentivirus-delivered stable gene silencing by RNAi in primary cells. RNA. 2003;9:493–501. 10.1261/rna.2192803 PMC1370415 12649500

[25] Li L, Sun L, Gao F, Jiang J, Yang Y, Li C, Gu J, Wei Z, Yang A, Lu R, Ma Y, Tang F, Kwon SW, Zhao Y, Li J, Jin Y. Stk40 links the pluripotency factor Oct4 to the Erk/MAPK pathway and controls extraembryonic endoderm differentiation. Proc Natl Acad Sci U S A. 2010;107:1402–7. 10.1073/pnas.0905657107 PMC2824367 20080709

[26] Jin Y, Xu XL, Yang MC, Wei F, Ayi TC, Bowcock AM, Baer R. Cell cycle-dependent colocalization of BARD1 and BRCA1 proteins in discrete nuclear domains. Proc Natl Acad Sci U S A. 1997;94:12075–80. 10.1073/pnas.94.22.12075 PMC23707 9342365

[27] Zhang Z, Liao B, Xu M, Jin Y (2007). Post-translational modification of POU domain transcription factor Oct-4 by SUMO-1. FASEB J.

[28] Lee HJ, Hinshelwood RA, Bouras T, Gallego-Ortega D, Valdes-Mora F, Blazek K, Visvader JE, Clark SJ, Ormandy CJ. Lineage specific methylation of the Elf5 promoter in mammary epithelial cells. Stem Cells. 2011;29:1611–9. 10.1002/stem.706 21823211

[29] Koh KP, Yabuuchi A, Rao S, Huang Y, Cunniff K, Nardone J, Laiho A, Tahiliani M, Sommer CA, Mostoslavsky G, Lahesmaa R, Orkin SH, Rodig SJ, Daley GQ, Rao A. Tet1 and Tet2 regulate 5-hydroxymethylcytosine production and cell lineage specification in mouse embryonic stem cells. Cell Stem Cell. 2011;8:200–13. 10.1016/j.stem.2011.01.008 PMC3134318 21295276

[30] Giakoumopoulos M, Golos TG (2013). Embryonic stem cell-derived trophoblast differentiation: a comparative review of the biology, function, and signaling mechanisms. J Endocrinol.

[31] Bayarsaihan D (2002). SSDP1 gene encodes a protein with a conserved N-terminal FORWARD domain. Biochim Biophys Acta.

[32] Wu L (2006). Structure and functional characterization of single-strand DNA binding protein SSDP1: carboxyl-terminal of SSDP1 has transcription activity. Biochem Biophys Res Commun.

[33] Beddington RS, Robertson EJ (1989). An assessment of the developmental potential of embryonic stem cells in the midgestation mouse embryo. Development.

[34] Niwa H, Miyazaki J, Smith AG (2000). Quantitative expression of Oct-3/4 defines differentiation, dedifferentiation or self-renewal of ES cells. Nat Genet.

[35] Niwa H, Toyooka Y, Shimosato D, Strumpf D, Takahashi K, Yagi R, Rossant J. Interaction between Oct3/4 and Cdx2 determines trophectoderm differentiation. Cell. 2005;123:917–29. 10.1016/j.cell.2005.08.040 16325584

[36] Rhee C, Lee BK, Beck S, Anjum A, Cook KR, Popowski M, Tucker HO, Kim J. Arid3a is essential to execution of the first cell fate decision via direct embryonic and extraembryonic transcriptional regulation. Genes Dev. 2014;28:2219–32. 10.1101/gad.247163.114 PMC4201284 25319825

[37] Vong QP, Liu Z, Yoo JG, Chen R, Xie W, Sharov AA, Fan CM, Liu C, Ko MS, Zheng Y. A role for borg5 during trophectoderm differentiation. Stem Cells. 2010;28:1030–8. 10.1002/stem.428 PMC2957878 20506138

[38] Kuckenberg P, Buhl S, Woynecki T, van Furden B, Tolkunova E, Seiffe F, Moser M, Tomilin A, Winterhager E, Schorle H. The transcription factor TCFAP2C/AP-2gamma cooperates with CDX2 to maintain trophectoderm formation. Mol Cell Biol. 2010;30:3310–20. 10.1128/MCB.01215-09 PMC2897582 20404091

[39] Hayashi Y, Furue MK, Tanaka S, Hirose M, Wakisaka N, Danno H, Ohnuma K, Oeda S, Aihara Y, Shiota K, Ogura A, Ishiura S, Asashima M. BMP4 induction of trophoblast from mouse embryonic stem cells in defined culture conditions on laminin. In Vitro Cell Dev Biol Anim. 2010;46:416–30. 10.1007/s11626-009-9266-6 PMC2862943 20033790

[40] Ng RK, Dean W, Dawson C, Lucifero D, Madeja Z, Reik W, Hemberger M. Epigenetic restriction of embryonic cell lineage fate by methylation of Elf5. Nat Cell Biol. 2008;10:1280–90. 10.1038/ncb1786 PMC2635539 18836439

[41] Kuckenberg P, Peitz M, Kubaczka C, Becker A, Egert A, Wardelmann E, Zimmer A, Brustle O, Schorle H. Lineage conversion of murine extraembryonic trophoblast stem cells to pluripotent stem cells. Mol Cell Biol. 2011;31:1748–56. 10.1128/MCB.01047-10 PMC3126346 21300784

[42] Rinehart J, Adjei AA, Lorusso PM, Waterhouse D, Hecht JR, Natale RB, Hamid O, Varterasian M, Asbury P, Kaldjian EP, Gulyas S, Mitchell DY, Herrera R, Sebolt-Leopold JS, Meyer MB. Multicenter phase II study of the oral MEK inhibitor, CI-1040, in patients with advanced non-small-cell lung, breast, colon, and pancreatic cancer. J Clin Oncol. 2004;22:4456–62. 10.1200/JCO.2004.01.185 15483017

