# Supplementary figures and images for: Single-stranded DNA binding protein Ssbp3 induces differentiation of mouse embryonic stem cells into trophoblast-like cells
Source: Stem Cell Res Ther. 2016 May 28;7:79. doi: 10.1186/s13287-016-0340-1 (PMC4884356; doi:10.1186/s13287-016-0340-1)

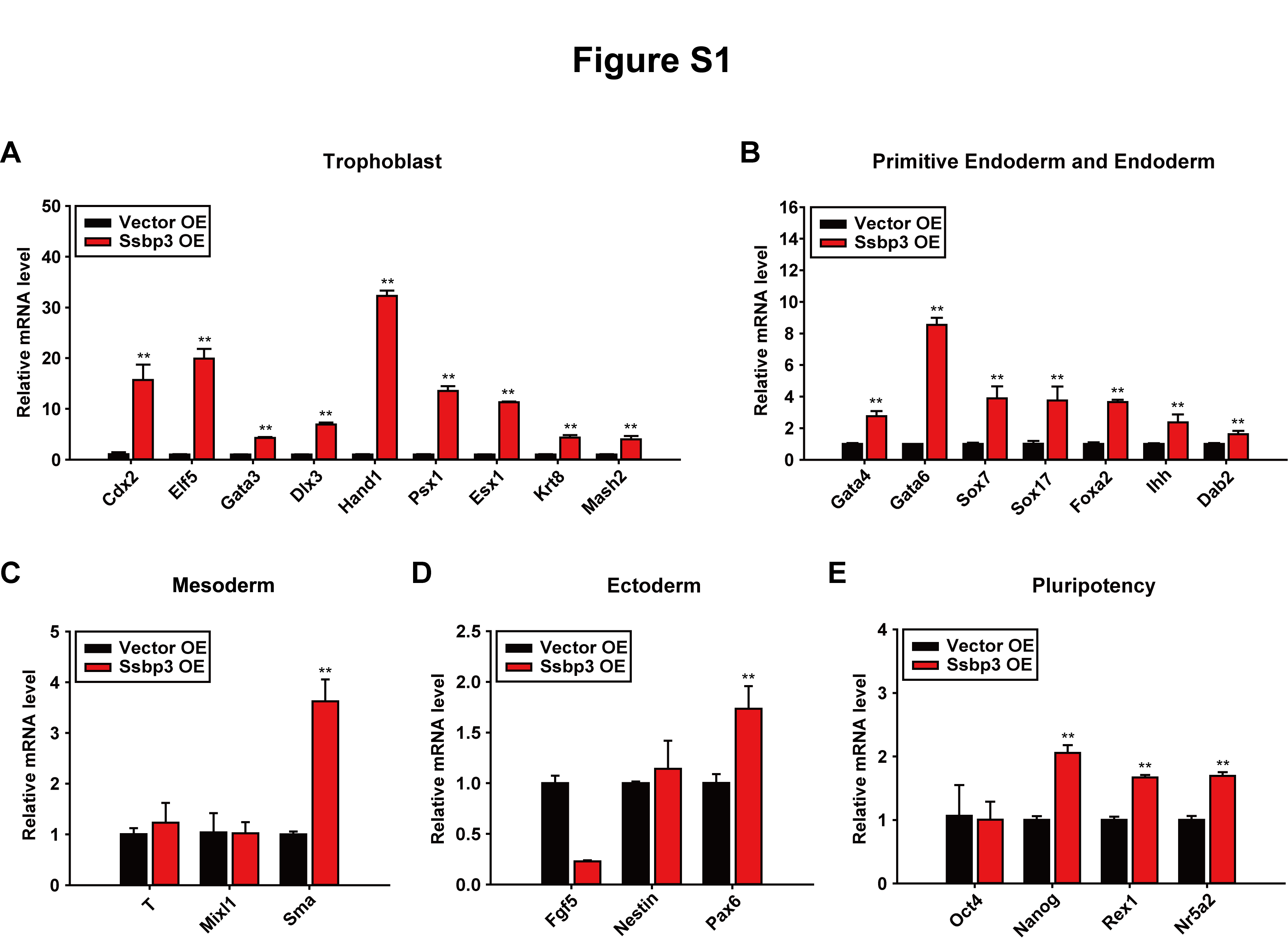

Supplement: Additional file 2: Figure S1. — Forced expression of Ssbp3 induces mouse ESC differentiation with a bias to trophoblast lineages under the LIF withdrawal condition. (A–E) Expression levels of pluripotency and lineage specific markers in ESCs overexpressing Ssbp3 were determined by qRT-PCR analyses, including markers for the trophoblast (A), primitive and definitive endoderm (B), mesoderm (C), ectoderm (D), and pluripotency (E). The average mRNA level in cells transfected with the control vector was set at 1.0. Data are shown as mean ± SD (n = 3). *p < 0.05, **p < 0.01. (TIF 306 kb) [file 13287_2016_340_MOESM2_ESM.tif]

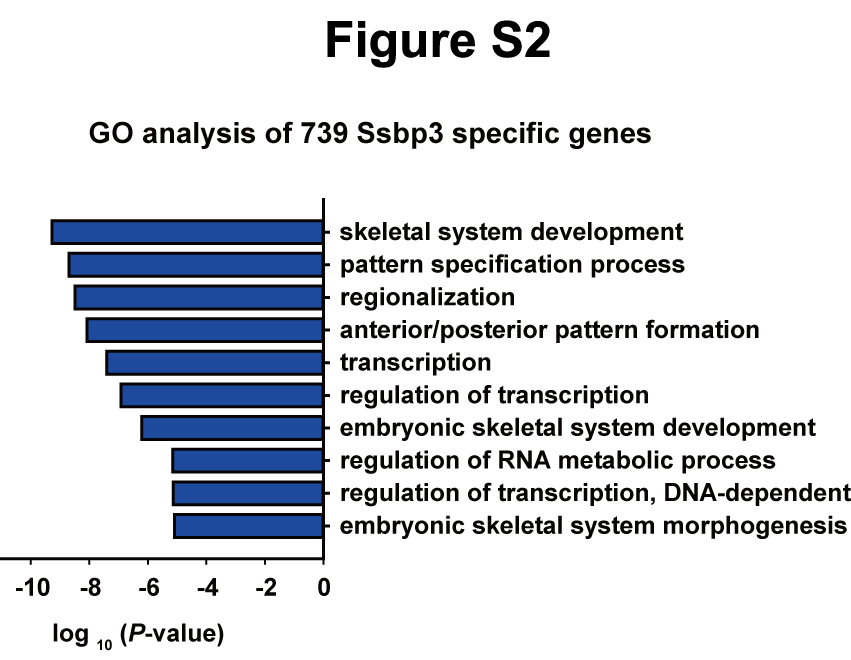

Supplement: Additional file 6: Figure S2. — GO analysis of 739 Ssbp3 specific genes compared with DEGs induced by Gata3 or Cdx2 overexpression. The significantly enriched GO terms are strongly related to embryonic skeletal system development and pattern specification process. (TIF 78 kb) [file 13287_2016_340_MOESM6_ESM.tif]

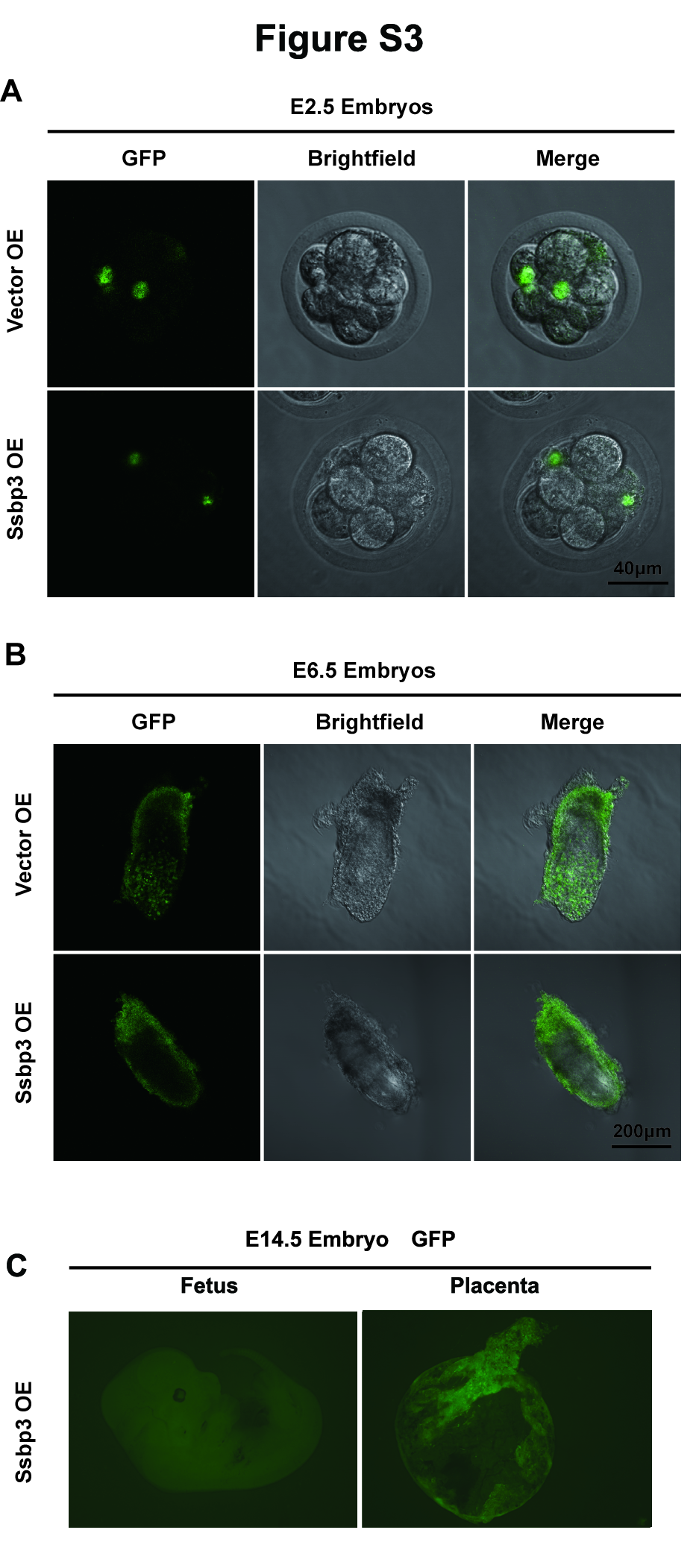

Supplement: Additional file 7: Figure S3. — Ssbp3-overexpressing ESCs mainly contribute to the placenta part in chimeric embryos. (A) GFP-labeled E14T cells overexpressing a vector or an Ssbp3 expression plasmid were injected into wild-type 8-cell-stage embryos. For each embryo, two GFP-labeled cells were injected. (B) Strong contribution of injected E14T cells overexpressing Ssbp3 to the extra-embryonic lineage in E6.5 chimeric embryos. Gross and fluorescence images of E6.5 chimeric embryos developed from embryos described in A. (C) E14T cells overexpressing Ssbp3 predominantly contributed to the placenta part of the E14.5 chimeric embryo. (TIF 7678 kb) [file 13287_2016_340_MOESM7_ESM.tif]
